# O11 *Neisseria Meningitidis* as a cause of isolated bilateral polyarticular native knee joint septic arthritis

**DOI:** 10.1093/rap/rkab067.010

**Published:** 2021-10-19

**Authors:** Saad Ahmed, Tom Walton, Adaeze Ugwoke, Freda Sundram, Ray Borrow

**Affiliations:** 1 Colchester Hospital, Colchester, United Kingdom; 2 Public Health England, Manchester, United Kingdom

## Abstract

**Case report - Introduction:**

Septic Arthritis is a medical emergency with a significant mortality and morbidity. The aim of management is to minimise the risk of irreversible joint damage and to preserve function.

We present the case of a 63-year-old lady admitted with bilateral knee pain and swelling, lower limb rash and a fever who was initially managed as a reactive arthritis but subsequent Polymerase Chain Reaction (PCR) molecular analysis revealed capsular group B N. meningitidis in bilateral knee aspirates.

We discuss the diagnostic challenges in differentiating septic arthritis from inflammatory arthritis, and the role of PCR molecular analysis in that process.

**Case report - Case description:**

A 63-year-old female presented with a 5-day history of painful, stiff and swollen knees bilaterally with decreased range of movement. Five days prior to presentation she suffered from a sore throat, fever and lower limb rash for which she was started on flucloxacillin with some improvement.

Co-morbidities included hyperlipidaemia and a hysterectomy. Regular medication included naproxen.

On examination her temperature was 37.7C and she was haemodynamically stable. Both knees demonstrated active synovitis. Admission bloods were remarkable for a CRP of 399 and deranged liver function tests.

Knee X-rays revealed a moderate effusion in the left knee, and a large effusion in the right.

The patient was given a dose of intravenous Co-amoxiclav with a working diagnosis of septic arthritis.

Aspiration of both knees was performed with 120 mls of yellow-coloured fluid aspirated from the left knee and 90 mls from the right knee. No organisms were identified on Gram Stain and no growth at 48 hours on culture. Synovial fluid from both knees was sent for broad-based bacterial 16S rDNA PCR molecular testing.

The patient was reviewed 4-weeks later at which stage her symptoms had improved. Prednisolone had been stopped a week prior with no deterioration in her joint symptoms.

Peripheral blood cultures taken during admission revealed no growth at 5 days.

After discharge the results of the molecular PCR testing on the synovial fluid samples became available and were positive for bacterial 16S rDNA. Referral to the Public Health England Meningococcal Reference Unit ensued and further molecular typing confirmed N. meningitidis with capsular genogroup B.

Subsequent non-culture sequencing of the factor H binding protein and PorA epitope revealed it to belong to the subtype P 1.12-1,9 and hence the utility of vaccination with Bexsero could not be determined.

**Case report - Discussion:**

The patient was unusual in having a primary meningococcal arthritis (PMA), defined as an acute septic arthritis without meningitis. The fever and transient rash may have suggested mild meningococcaemia. Direct bacterial invasion of the synovium via the bloodstream is likely to be the route of spread in our patient.

Confirming meningococcal disease used to be very challenging with negative peripheral blood culture results confounded by early antibiotic usage and the presence of fastidious and uncultivable organisms. Culture detection of N. meningitidis is 100% specific but is limited by a sensitivity of around 31%. After parenteral antibiotic administration the isolation rate of N. meningitidis drops from 50% to less than 5%.

The development of quantitative PCR (qPCR) has improved detection rates and a positive result is diagnostic. qPCR is a culture-independent assay that can quantify bacterial load, is easy to use and has flexibility in design.

The broad coverage of qPCR assay is able to use the 16S rRNA gene as a target and hence the overall result of the assay will be able to give both qualitative and quantitative characterisation. The qPCR is a nucleic acid amplification test and hence doesn’t require the presence of viable bacteria for a positive result; and hence the results are not affected by prior antibiotic administration.

The sensitivity of PCR has been shown to be higher than blood cultures with values of 47% and 31%, respectively; specificities of PCR have been noted to be above 96%. It has been previously reported that 31% of culture-negative but clinically suspected meningococcal disease cases were subsequently found to be blood PCR-positive. Comparing this with analysis of synovial fluid which has a gram stain sensitivity of between 29% and 50% and a culture sensitivity of up to 76% shows a marked improvement.

**Case report - Key learning points:**

Our case highlights the challenges in differentiating septic arthritis from a reactive arthritis in a patient presenting with knee pain, swelling and pyrexia who subsequently had N. meningitidis identified via 16S rDNA PCR testing.

Emphasis is placed on recognising polyarticular septic arthritis as a clinical entity with early joint aspiration being the priority of care. Polyarticular septic arthritis accounts for an estimated 15% of all cases of septic arthritis with a mean of three affected joints. Mortality rates in monoarticular septic arthritis have been estimated to be around 11%, rising to 50% for polyarticular disease.

In our patient the timeline of events is an important factor. It has been reported that patients with septic arthritis typically have symptoms for less than 2 weeks at presentation, with the characteristic features of hot, swollen, painful and restricted joints whereas a reactive arthritis presents 2 to 4 weeks after the preceding infection. Our patient suffered from a sore throat, presumed streptococcal in origin, 5 days prior to her oligoarticular symptoms, which led to admission.

It would also be pertinent to consider the relevance of the sore throat 5 days prior to presentation. It has been suggested that a post-streptococcal reactive arthritis is a distinct clinical entity from other forms of reactive arthritis. The peak incidence, age, pattern of joint involvement, extra-articular manifestations and HLA B27 association have been reported to vary between the aforementioned conditions and no causal role has been found for streptococcal throat infection.

In conclusion, we have described the first case in the literature of N. meningitidis being identified as the causative organism by PCR assay of synovial fluid in a patient with bilateral septic arthritis of the native knee joint.

